# Comment on Huijghebaert et al. Does Trypsin Oral Spray (Viruprotect^®^/ColdZyme^®^) Protect against COVID-19 and Common Colds or Induce Mutation? Caveats in Medical Device Regulations in the European Union. *Int. J. Environ. Res. Public Health* 2021, *18*, 5066

**DOI:** 10.3390/ijerph20010630

**Published:** 2022-12-30

**Authors:** Glen Davison

**Affiliations:** School of Sport and Exercise Sciences, Division of Natural Sciences, University of Kent, Canterbury CT2 7PR, UK; g.davison@kent.ac.uk

I read with interest the recent paper of Huijghebaert et al. published recently in this journal [1]. However, I must highlight a number of mistakes and inaccuracies in their interpretation and reporting of our study on the effects of Coldzyme^®^ Mouth Spray on upper respiratory tract infection symptoms in endurance athletes under free living conditions [2]. I have detailed these mistakes further below and respectfully request that these errors be corrected by Huijghebaert and colleagues.

The authors appear to have made several mistakes and inaccuracies in the interpretation of our data, and thus the conclusions they arrive at based on our data. The most significant of which being that they reported a result incorrectly, as follows: they stated that our data on “rescue medication” used as “debunking the spray’s benefits for treatment of common cold” was higher in the treatment group (48% vs. 38%). However, these results are actually the wrong way around, as in our study, it was 38% in the treatment group vs. 48% in the control. As such, their statement is not correct, and if they wish to use this data for such a conclusion, it should be noted that this would actually be in the opposite direction—i.e., it would support the claims for the treatment of the common cold. This also seems to be a biased focus on this particular measure (rescue medication) when there are more valuable data in the symptom data (Jackson score results); however, the authors seem to have ignored these results. The symptom data (Jackson scores) clearly show lower symptoms scores and a shorter episode duration in the treatment group. I have highlighted this and other areas where similar misinterpretations have been made in the paper of Huijghebaert et al. [1], and I hope that the authors will acknowledge these mistakes and correct them accordingly.

The authors make the following statements (below which I have noted the issues identified with their interpretations):

1. **Statement in paper:***In a smaller open-label study in athletes, the proportions of virus-protected persons per treatment group were not released, but there was no significant difference in number of CC episodes/person between the CZ-MD and control group.*
  **Problems with statement:** We are particularly confused by this statement. First of all, it seems to ignore the fact that ColdZyme (CZ-MD) was not used for prophylaxis in this study, and in fact this is clearly stated in the methods. That is, in the Treatment section, we wrote “Participants in the treatment (ColdZyme) group were asked to use the ColdZyme product in accordance with manufacturer instructions **for treatment of suspected common cold/URTI (at first self-perceived signs of URTI)**”. … So, this design does not allow for a comparison of “virus-protected persons” as it specifically investigates URTI treatment after the first symptoms begin to emerge (i.e., not prophylactic “protection”). Nevertheless, the incidence rates remain useful in this context to confirm similar exposure and risk of contracting URTI in the first place (before treatment) between groups. This leads to the second point here, the number of episodes per person is indeed reported in our paper, both in Table 1 and in the accompanying results description, as follows: Results section, “At least one URTI episode was recorded during the study period in 76.4% of all participants (77.0% control, 75.8% ColdZyme). In total 130 episodes were recorded by all participants over the study period with no difference between groups in the incidence rate (mean incidence rate per person over study period was: 1.1 ± 0.9 Control, 1.0 ± 0.8 ColdZyme, P = 0.290)”.
  Thus, the above statement by the authors appears to misrepresent our paper.
2. **Statement in paper:***The study mixing (unweighted) data obtained from winter seasons over 2 years claimed a reduction of 3.5 days with CZ-MD vs. the controls.*
  **Problems with statement:** This statement ignores the fact that participants were (A) stratified before randomization and (B) randomization was balanced at each site in each tranche, as described in the paper, as follows: “Participants were stratified by sport type, sex, age, and usual training volume and randomised to either control or ColdZyme group by random number generation (www.randomization.com). Randomisation was also applied at each site independently so that groups were matched within each location”.
3. **Statement in paper:***However, patients in the control group felt less need for using OTC medication than in the CZ-MD group (38% vs. 48%, not significant), undercutting the relevance of the findings.*
  **Problems with statement:** This statement in incorrect. The authors have the numbers the wrong way around here. As can be seen in the Results section of the paper, OTC use was 48% in the control and 38% in CZ-MD. See Medication section: “Participants felt the need to use OTC medication for 48% of cases (32/67 episodes) in Control and 38% of cases (24/63 episodes) in ColdZyme”.

The authors then use this data to cast doubt over the results we obtained, and continue with further statements such as “*OTC-rescue medication was felt needed in more CC episodes on CZ-MD than in the control group, debunking the spray’s benefits for treatment of CC in one study, while another open prospective study claims 23% reduced use of rescue medication with CZ-MD further fueling inconsistent findings” and in Table 2 “are not corroborated by the fact that less subjects in the control group felt need for rescue medication than on CZ-MD (38% vs. 48%)*”.

First of all, it is troubling that Huijghebaert and colleagues would make such a strong conclusion based only on these OTC data, which are a weak and indirect indication of illness symptoms (i.e., there are many reasons athletes may to choose to use OTC or not when they are ill), while ignoring the primary outcomes of the URTI reporting data, in which all symptoms scores and durations were lower in the treatment group. It is also troubling that they did this with incorrect data as the rates were actually the opposite way around to what the Huijghebaert et al. havereported here. According to the logic used by these authors, their first statement of “*further fueling inconsistent findings*” is incorrect, as both of the studies to which they refer are consistent in the direction of their findings. In addition, their second statement of “…*are not corroborated by the fact that less subjects in the control group felt need for rescue medication than on*” is incorrect, and according to the logic they applied here, the opposite would be true, in that these findings are corroborated by this. However, regardless of how the OTC data are interpreted, it is somewhat moot and also totally ignores the most relevant study outcome in relation to symptoms for URTI reporting. In the Jackson Score section of the results, it is clear that “*Overall Control* vs. *ColdZyme mean Jackson symptom score was 6.9 ± 2.8 in Control and 5.5 ± 2.4 in ColdZyme (P = 0.006)*”, as also reported in Table 1. We are thus puzzled as to why the authors have chosen to focus on a non-direct outcome such as OTC use to support this conclusion (although it does not actually support them anyway, as they have the values the wrong way around), yet ignore the above fact that the symptom scores were significantly lower with CZ-MD. Again, this is a misrepresentation of the study findings.

4. **Statement in paper:***Moreover, the reduction in training load during CC, the return to normal training and the total number of training days were not significantly different between the groups.*
  **Problems with statement:** This misrepresents the data in order to support their above conclusion, which is incorrect. It also ignores the fact that the duration of time with symptoms (and hence with reduced training) was longer in the control, and so the overall impact on ability to train was improved in the CZ-MD group. The way that Huijghebaert and colleagues use the data we report, on “return to normal training after symptoms had resolved’, is misleading because it ignores the fact that the total time out/lost was lower in CZ-MD, by an average of 3.5 days (as reported in the paper).

**In Table 2: the following statements are made:**

1. **Statement in table: *CZ-MD = Control group*** *for the number of episodes/person (no data on the proportions of persons per treatment group).*
  **Problems with statement:** As already noted, this is not correct, as the number of episodes per person is indeed reported, as mentioned above. Thus, the above statement by the authors appears to misrepresent our paper.
2. **Statement in table:** *Other parameters (e.g., claimed reduction of the CC episode by 3.5 days with CZ-MD vs. controls) are not corroborated by the fact that less subjects in the control group felt need for rescue medication than on CZ-MD (38% vs. 48%).*
  **Problems with statement:** As already noted, this is not correct, and in fact the numbers are the opposite way around (i.e., 38% is CZ-MD and 48% in the control use rates). Thus, if the authors are going to make such a claim based on these values, they have their conclusion the wrong way around, and, by this logic, these numbers agree with the reduced symptom ratings etc., observed with CZ-MD. In addition, and regardless of OTC data, the symptoms scores were all reduced in the treatment group, which is not appropriately reflected in the way this study is represented and presented in this paper by Huijghebaert and colleagues. Table 2 also ignores the fact that this study was not a prophylaxis study (i.e., treatment only began after symptoms appeared, i.e., likely days after infection occurred).
3. **Statement in table:** *No significant effects on training-related outcomes. Data on CC episodes were not or not consistently corrected for compliance vs. baseline variables and different treatment periods.*
  **Problems with statement:** This appears to ignore the separate analyses that were performed to take compliance with manufacturer instructions for use (IFU) into account. Huijghebaert et al. appear, in their paper, to imply that compliance was ignored in the first phase of our study. This is incorrect. The analysis of compliance was applied to all data across both study periods. We noted extra reminders (to participants) that were added for the second phase to help improve the compliance levels. However, regardless of the phase, those with low compliance were separated from those with good compliance with IFU for additional and separate analyses (in addition to the analysis on the whole group without separation), from which we indeed observed better outcomes in those with good compliance. This is also detailed in the paper, for example in the Symptom duration section, we provide the following information: “*Nevertheless, these subjects/episodes (with poor IFU compliance) from both Tranches do provide a useful comparator group for some statistical comparisons, ….. When those randomised to ColdZyme but with poor compliance (Poor IFU comp) were separated from those with good compliance (Good IFU comp) the observed effect between Control and ColdZyme groups becomes even more evident (episode duration 10.4 ± 8.5 days in Control* vs. *6.9 ± 3.5 days in ColdZyme Good IFU comp, P = 0.004). Direct comparison between compliance groups also shows a significantly shorter episode duration with good compliance (episode duration 9.3 ± 4.5 days in ColdZyme poor IFU comp* vs. *6.9 ± 3.5 days in ColdZyme Good IFU comp, P = 0.040)*”.

To summarize, Huijghebaert and colleagues have misinterpreted a number of our findings and made a significant mistake in that they reported the OTC usage data from our study incorrectly (i.e., having the numbers the wrong way around/assigned to the wrong groups). This error seems to feed into some of their conclusions. As such, it would seem that the authors need to correct the mistakes made and any conclusions that they subsequently derived based on these interpretations.
